# A124 PERORAL ENDOSCOPIC MYOTOMY IS THE PREFERRED TREATMENT FOR PATIENTS WITH SYMPTOMATIC ZENKER’S DIVERTICULUM

**DOI:** 10.1093/jcag/gwac036.124

**Published:** 2023-03-07

**Authors:** M Hasan, G Buduhan, R Liu, L Tan, S Srinathan, B Kidane

**Affiliations:** 1 Gastroenterology; 2 Thoracic Surgery, University of Manitoba, Winnipeg, Canada

## Abstract

**Background:**

Zenker’s diverticulum (ZD) is a mucosal herniation at the pharyngoesophageal junction presenting with dysphagia, regurgitation and aspiration. While open surgical myotomy (OSM) is the conventional treatment option, select patients can undergo myotomy using endoscopic techniques.

**Purpose:**

Evidence on third-space flexible endoscopy with myotomy is lacking, especially in North America. We aimed to assess the safety and efficacy of peroral endoscopic myotomy (POEM) for symptomatic ZD.

**Method:**

Retrospective cohort study was performed of consecutive patients undergoing OSM and POEM completed in a tertiary hospital from 2010-2020. Only patients with accessible electronic medical records and at least 3 months of follow-up were included in this study. Data collected included: demographics, comorbidities, ZD characteristics, clinical and patient-reported outcomes.

**Result(s):**

14 patients underwent OSM and 18 patients underwent POEM. 10 of the patients undergoing POEM were considered for but were not able to get stapled endoscopic myotomy, the most common reasons were related to the technical limitations (ZD size too small, unable to hyper-extend neck, unable to position suspension laryngoscope). There were no significant differences between groups in age (p=0.35), BMI (p=0.38), Charlson comorbidity index (p=0.26) and size of ZD (p=0.92). Length of stay was significantly lower for POEM (0.4 vs. 3.1, p<0.01). Complications were more common and severe with OSM (36%, n=5) versus POEM (11%, n=2). The only complications post-POEM were contained esophageal perforations. Complications post-OSM included esophageal perforations requiring open cervical drainage, surgical site infections, recurrent laryngeal nerve injury/paresis, esophageal strictures requiring multiple dilatations. All patients undergoing POEM had 100% technical success with post-treatment barium esophagogram showing 100% resolution of obstruction/hang-up. The median follow-up time post-POEM was 11 months. Over the follow-up period there was a significant improvement of patient-reported outcomes, with a mean decrease of Eckardt score (4.7 to 1.1, p<0.001) and mean increase of Dakkak-Watson score (20.7 to 41, p<0.001). Only one patient had persistent ongoing symptoms of dysphagia post-POEM and was subsequently diagnosed with ineffective esophageal motility on high resolution manometry.

**Conclusion(s):**

POEM is a minimally invasive treatment option for ZD with high treatment success as well as reduced length of stay and complications. It is less invasive than OSM, more versatile than stapled endoscopic myotomy and is less prone to technical limitations.

**Please acknowledge all funding agencies by checking the applicable boxes below:**

None

**Disclosure of Interest:**

None Declared

